# Rikkunshito as a Therapeautic Agent for Functional Dyspepsia and its Prokinetic and Non-Prokinetic Effects

**DOI:** 10.3389/fphar.2021.640576

**Published:** 2021-06-08

**Authors:** Kazumi Inokuchi, Tatsuhiro Masaoka, Takanori Kanai

**Affiliations:** Division of Gastroenterology and Hepatology, Department of Internal Medicine, Keio University School of Medicine, Tokyo, Japan

**Keywords:** functional and motility disorders of the stomach, non-prokinetic treatment, rikkunshito, Kampo, functional dyspepsia

## Abstract

Prokinetics is one of the therapeutic agents for functional and motility disorders of the stomach. However, its efficacy is limited. Kampo medicine is a unique medical system that was developed in Japan. In Kampo medicine, herbal medicine is prescribed based on the patient’s condition. Therefore, even for functional and motility disorders of the stomach, some herbal medicines are considered as a therapeutic option. Recently, there has been an increase in evidence for the efficacy or the mechanism of herbal medicine for functional and motility disorders of the stomach. Among these, rikkunshito is a well-studied herbal medicine that could be used as an alternative to prokinetics. In this review, we discuss the possibilities of rikkunshito for functional dyspepsia with its prokinetic and non-prokinetic effects and provide an overview of their current use with a focus on their therapeutic mechanism.

## Introduction

Kampo is a traditional herbal medicine that was introduced from China to Japan. After that, Kampo medicine has been uniquely developed in Japan. Research on Kampo medicine has thus been studied mainly in East Asia and has not yet garnered worldwide study. This way of evaluating patients is different from Western medicine. The most characteristic aspect of Kampo medicine lies in how it is used to evaluate patients. It evaluates the whole body condition, called the “pattern” and “qi, blood, and fluid”, not each organ. Based on the patient’s condition, herbal medicine is prescribed in Kampo medicine. Therefore, its application is broad and can include patients with Functional Dyspepsia (FD). Some herbal medicines are prescribed to patients with FD, and, of these, rikkunshito is a well-studied herbal medicine. This review focuses on rikkunshito and discusses its potential as an alternative therapeutic option for FD besides traditional treatments.

## Pathophysiology and Treatment of FD

Functional gastrointestinal disorders (FGIDs) are characterized by subjective abdominal symptoms and lack of organic disease. FGIDs are defined by the Rome IV criteria, which were revised in 2016 (Drossman and Hasler, 2016). Functional dyspepsia (FD) is one of the FGIDs of gastroduodenal lesions. In the Rome IV criteria, symptoms of FD are bothersome postprandial fullness, early satiation, epigastric pain, and epigastric burning occurring more than 6 months prior to diagnosis and persisting for more than 3 months. FD is sub-classified as post-prandial distress syndrome (PDS) or epigastric pain syndrome (EPS), and these sub-classifications can overlap.

The pathophysiology of FD is heterogeneous, and it involves various pathological conditions, such as dysmotility, visceral hypersensitivity, low-grade inflammation, dysregulation of the gut-brain axis, and psychological factors. Recently, the involvement of impaired duodenal mucosal integrity has been suggested (Vanheel et al., 2014; Wauters et al., 2020), although the details of the mechanism have not been fully elucidated.

Because of the heterogeneity in pathophysiology, the basic treatment of FD has not yet been established. When FD is diagnosed, the presence of a *Helicobacte* *r pylori* infection should be checked for and, if present, treated. If an infection is absent or symptoms do not improve after treatment, proton pump inhibitors (PPI) and H_2_ receptor blockers, or prokinetics such as acotiamide, domperidone, mosapride, and itopride, should be used. If no improvement is observed, additional antidepressants are recommended (Moayyedi et al., 2017).

The usefulness of PPI is constantly discussed. Some reports indicated that a 2−8-week dosing is more useful than placebo (Pinto-Sanchez et al., 2017), while others suggested that it is useful when complicated by gastroesophageal reflux disease (GERD) and is ineffective on FD itself (Wong et al., 2002; Pinto-Sanchez et al., 2017; Tack and Camilleri, 2018), and the efficacy of PPI is thus controversial. Although PPI are generally considered safe and well tolerated, they are associated with a risk of developing a *Clostridium difficile* infection, pneumonia, fractures, and acute interstitial nephritis with long-term administration (Wilhelm et al., 2013).

Although the effectiveness of prokinetics for FD was analyzed in the meta-analysis, there is a variation in the report (Moayyedi et al., 2017), and the evidence level is not high. Thus, a high-quality randomized controlled trial (RCT) is desirable (Pittayanon et al., 2018).

FD is not a life-threatening disease; however, it remarkably burdens the quality of life. Therefore, establishing effective therapy is urgently needed.

## What is Rikkunshito?

Rikkunshito is an herbal medicine that is widely used for improving gastrointestinal symptoms, such as FD, mainly in Asian countries. In China, it is called Liu-Jun-Zi Tang, and it is a Chinese herbal formula originally described in the Chinese classic medical book.

In Kampo medicine, when prescribing herbal medicine, the condition of patients is often assessed using the labels “pattern” and “qi, blood, and fluid.” This concept markedly differs from that of Western medicine. In brief, the “pattern” is the evaluation of physical fitness and resistance to illness, and “qi, blood, and fluid” considers the causes of the disorder in these three parts. One way of dividing the “pattern” is “excess or deficiency.” Individuals who are thin, delicate, and prone to diarrhea and chilling and those with weak gastrointestinal function a low stamina are classified as having a deficiency. Conversely, those who have sufficient energy and muscular strength are classified as having an excess pattern. Rikkunshito is prescribed mainly for patients with a deficiency pattern. When translating rikkunshito directly to English, it would be translated to “soup of six nobles.” Rikkunshito is composed of the following eight herbal medicines: extracts of *Atractylodes lancea* Rhizome, Ginseng, *Pinellia tuber*, *Poria sclerotium*, *Jujube*, *Citrus unshiu* Peel, *Glycyrrhiza*, and *Ginger*. Among these, the extracts of the *Atractylodes lancea* Rhizome, *Ginseng*, *Poria sclerotium*, *Pinellia tuber*, *Citrus unshiu* Peel, and *Glycyrrhiza* were imagined as the six nobles (Yagi et al., 2004). It has been clarified that the ingredients of rikkunshito include β-eudesmol derived from the *Atractylodes lancea* Rhizome, ginsenosides derived from the Ginseng, hesperidin derived from the *Citrus unshiu* Peel, glycyrrhizin derived from the *Glycyrrhiza*, and shogaol derived from the ginger. Rikkunshito is widely used for upper gastrointestinal disorders in Japan. As side effects, hepatic dysfunction or pseudoaldosteronism owing to glycyrrhizin contained in *Glycyrrhiza* are known.

### Clinical Effect of Rikkunshito on FD

In 1993, Tatsuta et al. (Tatsuta and Iishi, 1993) divided 42 patients with FD into the rikkunshito group (*N* = 22) and the placebo group (*N* = 20) and compared gastric emptying using the acetaminophen absorption method. They reported that gastrointestinal symptoms significantly improved and gastric emptying was enhanced in the rikkunshito group after 7 days of medication. In 2014, Suzuki et al. performed a double-blind RCT, dividing FD patients into a rikkunshito group and a placebo group where patients received 8 weeks of medication. Improvement of epigastric pain (*p* = 0.04) and postprandial fullness (*p* = 0.06) were observed in the group treated with rikkunshito, and this suggested that the efficacy may be lower in *H. pylori*-uninfected individuals (Suzuki et al., 2014). In a post hoc analysis (Togawa et al., 2016), it has been reported that a lower level of ghrelin in blood was an independent factor supporting the effectiveness of rikkunshito in FD patients without *H. pylori* infection and that it was more therapeutically effective in patients with *H. pylori* infection in the absence of alcohol consumption. Another double-blind, RCT was conducted in Japan in which 192 FD patients who met the Rome III criteria were enrolled (Tominaga et al., 2018). After a 2-week single-blind placebo period, patients were divided into the rikkunshito group (*N* = 64) and the placebo group (*N* = 61) for 8 weeks. The authors showed significant improvement in psychiatric symptoms and upper gastrointestinal symptoms in the rikkunshito group (Tominaga et al., 2018). However, a systematic review and a meta-analysis review have suggested that further studies are needed for supporting the usefulness of rikkunshito (Hoshino et al., 2019). Mausy et al. examined the effect of rikkunshito on symptoms and gastric motility in European FD-PDS patients using a randomized, placebo-controlled, crossover study. Rikkunshito did not alter gastric motility. Treatment with rikkunshito improved upper gastrointestinal symptoms in FD patients, but similarly, high placebo effects were observed (Masuy et al., 2020). A meta-analysis has reported that the combination of Western medication and rikkunshito was more helpful than the use of Western medication alone (Ko et al., 2020). Further accumulation of evidence for the clinical effect of rikkunshito on FD is demanded.

## Mechanism of Action of Rikkunshito on Sensory-Motor Function in Animals *via* THE NON-Ghrelin Mediated Pathway

When the effects of metoclopramide, trimebutine, cisapride, and rikkunshito were examined on the gastric adaptive relaxation in guinea pig stomachs, only rikkunshito induced adaptive relaxation of the stomach and improvement of the gastric volume. Oral administration of N(G)-nitro-L-arginine (L-NNA), a nitic oxide (NO) synthetic inhibitor to the stomach, inhibited gastric adaptive relaxation. Rikkunshito, but not gastroprokinetics, overcame the effect of the NG-nitro-L-arginine. This result suggests the action of rikkunshito *via* the NO-mediated pathway (Hayakawa et al., 1999).

Serotonin (5-hydroxytryptamine (5-HT)) is synthesized from tryptophan, and 90% of 5-HT exists in the gastrointestinal tract. 5-HT regulates the sensitization, motility, and secretion of the gastrointestinal tract. 5-HT receptors are classified into seven families and 14 subtypes. Among these, 5-HT_3_ and 5-HT_4_ receptors are involved in the regulation of sensation and motor function in the gastrointestinal tract (Grider et al., 1998; Taniyama et al., 2000), and 5-HT_2C_ is involved in eating behavior.

The efficacy of rikkunshito for cisplatin-induced anorexia in rats has been reported. Administration of 5-HT and dopamine-induced delayed gastric emptying in rats. Administration of a 5-HT_3_ receptor antagonist, ondansetron, and rikkunshito did not improve the delayed gastric emptying induced by dopamine but ameliorated the delayed gastric emptying induced by 5-HT. Furthermore, their effect was abolished by atropine administration. Therefore, the 5-HT_3_ receptor-mediated pathway has been suggested as a mechanism of action of rikkunshito (Tominaga et al., 2011). Additionally, several flavonoids contained in rikkunshito cross the blood-brain barrier. The action of rikkunshito for anorexia may be mediated via 5-HT_2C_ receptors in the central nervous system (Arai et al., 2013). Moreover, association between plasma ACTH and cortisol levels under stress and rikkunshito is also reported (Naito et al., 2003).

Regarding gastric emptying, the effect of rikkunshito is mainly studied with rats. Improvement of delayed gastric emptying *via* the NO-mediated pathway is reported. In this study, as ingredients in rikkunshito, roles of Hesperidin in *Citrus unshu* Peel and L-Arginine in Ginseng, *Pinella ternate* and *Atractylodes lancea* Rhizome are suggested (Kido et al., 2005). Improvement of delayed gastric emptying *via* antagonistic action of the 5-HT(3) receptor is suggested (Sadakane et al., 2011). Moreover, potentiation of phase III-like contractions by rikkunshito is reported. In this study, as ingredients in rikkunshito, roles of Hesperidin in *Citrus unshu* Peel, and L-Arginine in Ginseng, *Pinella ternate* and Atractylodin in *Atractylodes lancea* Rhizome are suggested (Nahata et al., 2018). Regarding gastric adaptive relaxation or gastric accommodation, the effect of rikkunshito is mainly studied with guinea pigs. Potentiation of gastric adaptive relaxation is reported (Hayakawa et al., 1999). Moreover, suppression of impairment of gastric accommodation *via* the NO- and 5-HT(2B) receptor-mediated pathway is reported (Miwa et al., 2016). These findings are summarized in Table 1.

**TABLE 1:** List of *in vivo* studies that examine the efficacy of rikkunshito on motor patterns.

Effect	Ingredient	Mechanism	Utilized animals	References
Improvement of delayed gastric emptying	Hesperidin, L-Arginine	NO mediated action	Rats	Kido et al. (2005)
Improvement of delayed gastric emptying		Antagonistic action of the 5-HT(3) R	Rats	Tominaga et al. (2011)
Potentiate phase III-like contractions	Atractylodin		Rats	Nahata et al. (2018)
Potentiate gastric adaptive relaxation			Guinea pigs	Hayakawa et al. (1999)
Suppress impairment of gastric accommodation		NO and 5-HT(2B)R mediated pathway	Guinea pigs	Miwa et al. (2016)

NO, nitric oxide; 5-hydroxytryptamine 3 receptor, 5-HT(3) R; 5-hydroxytryptamine 2B receptor, 5-HT(2B)R.

In 2020, Zhao et al. reported that rikkunshito significantly attenuated visceral hypersensitivity in FD model rats, and the overexpression of EC cells, 5HT, TPH1, PAX4, and 5HT3R in the duodenum of FD model rats was also reduced by rikkunshito decoction (Zhao et al., 2020).

## Mechanism of Action of Rikkunshito *via* the Ghrelin-Mediated Pathway

Ghrelin is a 28-amino-acid peptide identified as a ligand for the growth hormone secretagogue receptor (GHS-R) in 1999. Ghrelin is mainly secreted by gastric endocrine X/A-like cells (Kojima et al., 1999). Secreted ghrelin enhances food intake through the activation of food intake stimulating neuropeptide Y (NPY)/agouti-related protein (AgRP) neurons and the inhibition of anorexigenic pro-opiomelanocortin (POMC) neurons. These neurons exist in the arcuate nucleus of the hypothalamus (Kageyama et al., 2010; Andrews, 2011). Ghrelin regulates growth hormone secretion and feeding, and the blood level of ghrelin is negatively correlated with the body mass index in healthy individuals and uncomplicated type 2 diabetes mellitus patients; it has been reported to be lower in obese patients and higher in lean patients with cancer, chronic heart failure, and anorexia nervosa (Ueno et al., 2007). Administration of a selective serotonin uptake inhibitor containing fenfluramine reduced blood ghrelin levels and gastrointestinal peristalsis in rats. Oral administration of rikkunshito improved blood ghrelin levels and dietary intake (Fujitsuka et al., 2009). In clinical studies in humans, the administration of rikkunshito increases blood ghrelin levels in healthy participants and in patients after gastrectomy (Fujitsuka and Uezono, 2014). Rikkunshito may ameliorate anorexia by promoting the secretion of ghrelin. In 2008, Takeda et al. firstly reported the relationship between rikkunshito and ghrelin. They reported oral administration of rikkunshito suppressed the cisplatin-induced decrease in plasma acylated-ghrelin levels. (Takeda et al., 2008). Since then, a variety of rikkunshito’s ghrelin-mediated mechanisms have become apparent. Thirty-two ingredients were considered as typical in rikkunshito. In total, 18 or 21 ingredients from plasma or urine were detected after administration of rikkunshito in healthy volunteer study (Kitagawa et al., 2015). Among these, 9 ingredients were elucidated their mechanism as ghrelin mediated therapeutic effect of rikkunshito by *in vivo* and *in vitro* study. Pachymic acid in *Poria sclerotium* and 10-gingerol in *Ginger* inhibit ghrelin diacylation by inhibiting ghrelin-deacylating enzymes (Sadakane et al., 2011). Heptamethoxyflavone, Nobiletin, and Isoliquiritigenin in *Citrus unshu* Peel improve ghrelin resistance via central phosphodiesterase III inhibition (Takeda et al., 2010). Atractylodin in *Atractylodes lancea* Rhizome, Hesperidin in *Citrus unshu* Peel, 8-gingerol in *Ginger*, and Isoliquiritigenin in *Glycyrrhiza* promote ghrelin secretion (Fujitsuka et al., 2011; Nahata et al., 2013; Yamada et al., 2013). Particularly, Hesperidin and 8-gingerol promote ghrelin secretion by central 5-HT2C receptor antagonism, and Isoliquiritigenin promotes ghrelin secretion *via* peripheral 5-HT2B receptor antagonism. The effects of the above ingredients of rikkunshito are summarized in Table 2.

**TABLE 2:** Rikkunshito ingredients with a ghrelin-related effect.

Herb	Ingredient	Target	Role	References
*Poria sclerotium*	Pachymic acid		Inhibit ghrelin degration	Sadakane et al. (2011)
*Citrus unshu* Peel	Heptamethoxyflavone	PDEIII	Improve ghrelin resistance	Takeda et al. (2010)
	Nobiletin	PDEIII	Improve ghrelin resistance	Takeda et al. (2010)
	Isoliquiritigenin	PDEIII	Improve ghrelin resistance	Takeda et al. (2010)
	Hesperidin	5-HT(2C)R	Promote ghrelin secretion	Nahata et al. (2013)
*Ginger*	8-Gingerol	5-HT(2C)R	Promote ghrelin sectertion	Nahata et al. (2013)
	10-Gingerol		Inhibit ghrelin degration	Sadakane et al. (2011)
*Glycyrrhiza*	Isoliquiritigenin	5-HT(2B)R	Promote ghrelin secretion	Yamada et al. (2013)
*Atractylodes lancea* Rhizome	Atractylodin		Promote ghrelin secretion	Fujitsuka et al. (2011)

PDE III, phosphodiesterase III; 5-hydroxytryptamine 2C receptor, 5-HT(2C)R, 5-hydroxytryptamine 2B receptor, 5-HT(2B)R.

Most of the circulating ghrelin is synthesized and secreted by X/A-like cells in the stomach. Morevoer, neurons in the hypothalamus also express ghrelin (Castañeda et al., 2010). As mentioned above, rikkunshito has both of peripheral and central effect for promoting ghrelin secretion. However, whether the central or peripheral effect of rikkunshito is more important for promoting ghrelin secretion is not clarified yet.

The plasma levels of ghrelin decrease with increasing gastric mucosal atrophy (Suzuki et al., 2004). In the randomized clinical trial of rikkunshito for the treatment of functional dyspepsia, rikkunshito was relatively more effective among *Helicobacter pylori*-infected participants (rikkunshito: 40.0% vs. placebo: 20.5%, *p* = 0.07) and seemed less effective among *H. pylori*-uninfected participants (rikkunshito: 29.3% vs. placebo: 25.6%, *p* = 0.72). Among *H. pylori-positive* individuals, acyl ghrelin levels were improved just in rikkunshito group (Suzuki et al., 2014). From these findings, there is a possibility that rikkunshito returns the ghrelin level to normal when the ghrelin secretion is below the normal level.

A randomised double-blind cross-over study of healthy volunteers showed an increase in the appetite and energy consumption in the free-choice buffet (Wren et al., 2001). Therefore, long term administration of ghrelin may have a risk for evoking obesity. However, to the best of our knowledge, a risk of obesity has not yet been linked to rikkunshito administration.

## Discussion

As mentioned above, in an *in vivo* study, the mechanisms of action of rikkunshito, such as promoting ghrelin secretion, mediating gastric adaptive relaxation, and stimulating gastric emptying, have been elucidated (Kido et al., 2005; Arai et al., 2013; Fujitsuka and Uezono, 2014). The effect of rikkunshito on gastric motility in humans has been studied with various modalities. Kusunoki et al. measured the cross-sectional area of the proximal part of the stomach using abdominal ultrasound and calculated the gastric emptying rate, motility index, and reflux index before and after administration of rikkunshito in patients with FD. However, there was no improvement in symptoms; all measurements except the reflux index significantly increased after the administration of rikkunshito (Kusunoki et al., 2010). Shiratori et al. used a barostat for measuring the volume of the stomach before and after the administration of rikkunshito in nine healthy volunteers and reported that the volume of the stomach increased significantly after the administration, with improvement in perceived stress (Shiratori et al., 2011). Mausy et al. examined the effect of rikkunshito on intra-gastric pressure with high-resolution manometry before and after treatment with rikkunshito; however, rikkunshito did not alter gastric motility (Masuy et al., 2020). These differences might be caused by the characteristics of each modality. Regarding the effect of rikkunshito, *in vivo* studies were ahead of clinical studies. Compared with the results of the aforementioned *in vivo* studies, human studies did not entirely reproduce results of *in vivo* studies. Further research on the effects of rikkunshito on the human stomach and its association with symptomatic improvement is needed.

As described in the Introduction section, Kampo medicine, in contrast to Western medicine, a suitable drug for the patient is prescribed based on the assessment of the patient’s pattern in addition to the symptoms. Furthermore, herbal medicine contains a large number of active ingredients; the effects of herbal medicine come from intertwined ingredients and have a complex mechanism of action. They can act on the whole body rather than locally. Kampo medicine may not be very popular in Western countries because of the differences in the way of choosing herbal medicine. Herbal medicine used in Kampo medicine includes multiple herbals, and each herb includes many chemicals. Therefore, herbal medicine has various mechanisms, whereas a western drug is generally composed of a single chemical with a single mechanism of action (Yu et al., 2006).

Moreover, because many of the previous studies on Kampo medicine conducted in the 1980s–1990s have been published in Japanese, these pioneering studies have not been referred to in English literature; they have not been indexed in PubMed. This inaccessibility of studies may be one of the reasons for the difficulty in sharing the results globally (Oka et al., 2014). There is also a possibility that unexpected drug interactions may emerge in combination with Western medicines. Some side effects and components of herbal medicine, such as pseudoaldosteronism and glycyrrhizin in *Glycyrrhiza*, have been identified. Though it is not so for all cases, some drug interaction between herbal medicine and Western drugs could be solved through cautious use; therefore, the possibility of safe usage of Kampo in Europe is suggested (Homma, 2016).

**FIGURE 1:**
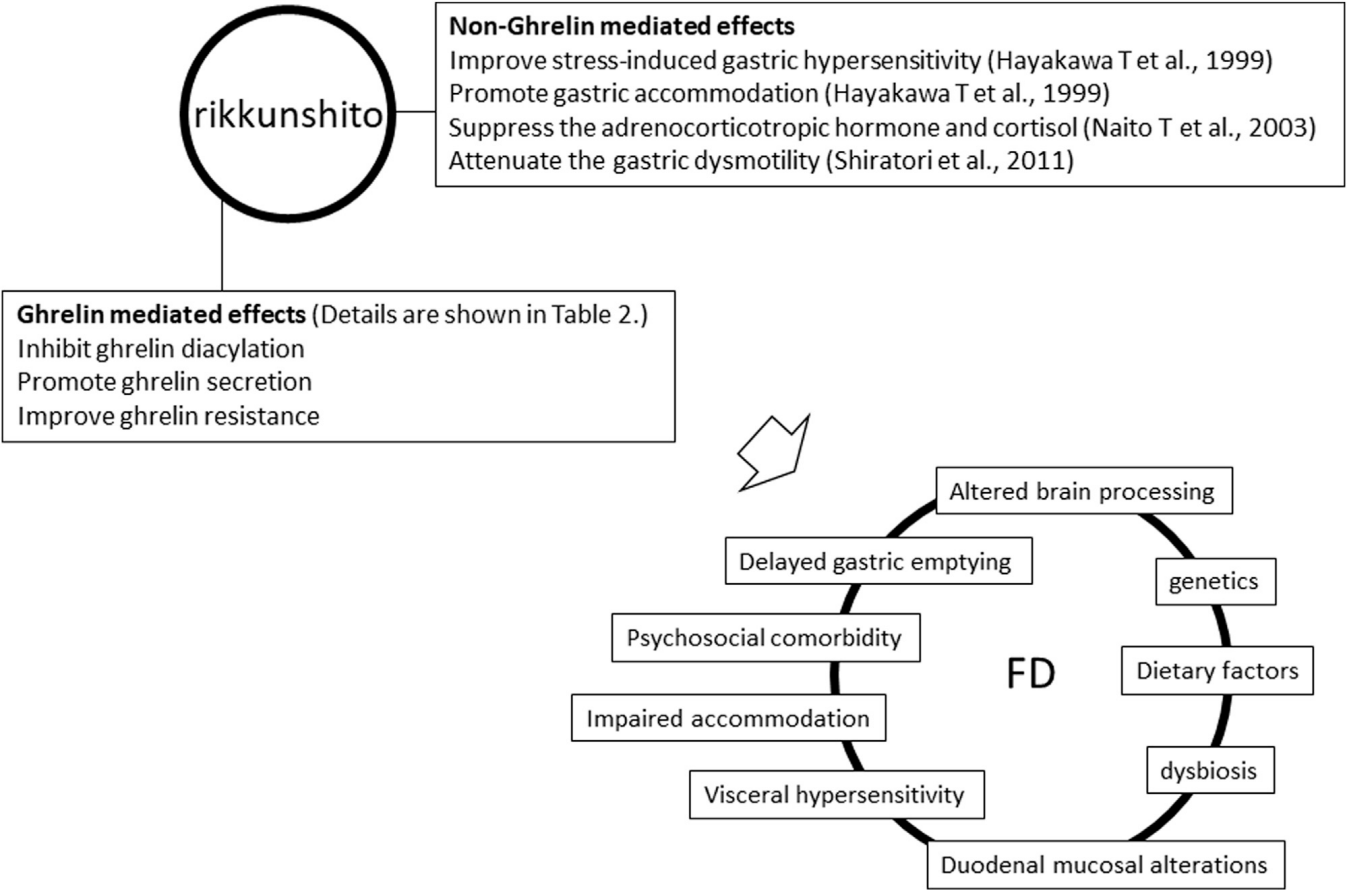
Multiple effects of rikkunshito and multiple pathophysiologies of functional dyspepsia.

As mentioned above, Rikkunshito can exert prokinetic activity and other effects, such as visceral hypersensitivity alleviation. There is no high-quality and sufficient evidence on the effectiveness of rikkunshito on FD. In contrast, it may be of sufficient value to attempt approaches applying Kampo medicine for complex and poorly understood pathologies, such as FD (Figure 1). In the future, we hope that Kampo, including rikkunshito, will become popular worldwide, and studies to elucidate the disease state of FD and the efficacy of Kampo will be actively performed.
